# Patterns of Telehealth Use Across the Cancer Care Continuum and Assessment of Patient and Geographic Factors Associated With Key Healthcare Outcomes: Retrospective Study

**DOI:** 10.2196/79956

**Published:** 2025-10-30

**Authors:** Kassandra I Alcaraz, Christopher Kitchen, Thomas Richards, Chintan J Pandya, Jonathan P Weiner, Elham Hatef

**Affiliations:** 1 Department of Oncology, Johns Hopkins University, 2024 E. Monument Street, Baltimore, MD, 21205, United States, 1 4109788006; 2 Department of Epidemiology, Johns Hopkins Bloomberg School of Public Health, Baltimore, Maryland, USA; 3 Sidney Kimmel Comprehensive Cancer Center, Johns Hopkins University, Baltimore, Maryland, USA; 4 Department of Health Policy and Management, Center for Population Health Information Technology, Johns Hopkins Bloomberg School of Public Health, Baltimore, Maryland, USA; 5 Division of General Internal Medicine, Department of Medicine, Johns Hopkins School of Medicine, Baltimore, Maryland, USA

**Keywords:** telehealth, cancer care, utilization patterns, geographic barriers, cancer-related health outcomes

## Abstract

**Background:**

Although the use of telehealth has declined since the pandemic, it remains a popular mode of care delivery across the cancer care continuum. Understanding telehealth in the context of cancer care is essential, as its benefits and challenges may differ among diverse population groups and geographic areas.

**Objective:**

This study aimed to examine patterns of telehealth utilization across the cancer care continuum and to identify factors associated with the receipt of telehealth in a large patient population. This study also aimed to assess the telehealth's impact on key health care delivery outcomes.

**Methods:**

We used an annualized retrospective cohort design using patient data from the Johns Hopkins Health System (JHHS), a large regional academic health center in Maryland. The study analyzed electronic health record (EHR) data covering the period from January 1, 2019, to December 31, 2023. Chronic conditions were defined through the Johns Hopkins Adjusted Clinical Groups (ACG) System, which identifies comorbidities based on the International Classification of Diseases, Tenth Revision, Clinical Modification, codes in the electronic health record. In addition, we used publicly available geospatial data (eg, internet connectivity, rural–urban commuting area) to assess telehealth receipt associations. Statistical modeling, including generalized estimating equations, was used to evaluate variations in telehealth utilization and outcomes.

**Results:**

A total of 124,974 adult patients receiving cancer-related care at Johns Hopkins Health System were identified during the study period. Telehealth users were significantly older (52.2% aged ≥65 years, 19,942 patients) compared to nonusers (48.7%, 42,209 patients). In addition, these users were more likely to be male (45.4%, 17,365 patients vs 40.2%, 34,839 patients) and to identify as White (70.8%, 27,071 patients vs 64.7%, 56,122 patients). Telehealth users also had a higher prevalence of comorbidities, with 61.5% (23,503 patients) reporting 3 or more chronic conditions compared to 38.0% (33,000 patients) among nonusers. A positive correlation was noted between rural–urban commuting area codes and telehealth service utilization (ρ=0.36; *P*<0.05), indicating higher usage in more rural areas. Conversely, average maximum download and upload speeds showed an inverse relationship with telehealth utilization (ρ=−0.22; *P*<0.05; and ρ=−0.34; *P*<0.05, respectively). Adjusted analyses indicated that concurrent telehealth use was associated with reduced odds of emergency department visits (0.916, 95% CI 0.884-0.948) and hospitalizations (0.830, 95% CI 0.799-0.863), acknowledging the potential influence of residual confounding.

**Conclusions:**

Telehealth has emerged as a crucial mode of care delivery for patients with complex conditions such as cancer. Understanding usage patterns and factors influencing telehealth across the cancer care continuum, including geographic barriers, is vital to optimizing its implementation and ensuring health care systems meet the diverse needs of patients with cancer in a value-based care environment.

## Introduction

### Background

The COVID-19 pandemic disrupted cancer care delivery, exacerbated existing disparities in cancer care, and may have a long-term detrimental impact on cancer mortality [1-4]. The pandemic also catalyzed the delivery of telehealth, a digitally supported, remote health care tool that has been available for decades but had limited adoption before the pandemic [5,6]. Although receipt of telehealth has declined since the beginning of the pandemic, telehealth remains a popular care delivery channel and continues to exceed prepandemic utilization levels [7,8]. In the postpandemic era, across the cancer care continuum, the successful integration of telehealth with traditional in-person care processes has critical implications for the provision of care for patients with different cancer-related acute and chronic conditions.

In recent years, a growing body of evidence has demonstrated the benefit of telehealth for multidisciplinary care across the cancer care continuum [9-14]. However, differences in the receipt of telehealth are documented among patient subpopulations, indicating significantly lower receipt among historically underserved populations and differential access across clinical settings [15,16]. For telehealth to be effective as a permanent stand-alone substitute or in combination with in-person cancer care, research is needed to identify the extent to which telehealth is an optimal mode of cancer care delivery for different clinical settings and patients with cancer [4]. This is especially important in cancer care, given the range of care settings, including medical, surgical, and radiation oncology, and for survivorship care, which may occur in nonspecialized settings, such as primary care. Specifically, research is needed on possible current differences in receipt of telehealth-based cancer care and the association of telehealth with outcomes such as health care utilization [17]. Thus, a greater understanding of telehealth is vital, especially because the benefits and barriers of telehealth, as with other digital health tools, may vary across different population groups [18,19].

### Objectives

Given the need to better understand telehealth in cancer care and associated outcomes, this study implemented a retrospective analysis to examine patterns of telehealth utilization across the cancer care continuum among patients with cancer in a large referral health care system in Maryland. It also examined factors associated with the receipt of telehealth and the association of telehealth with health care outcomes.

## Methods

### Design and Setting

In a retrospective study, we used the Johns Hopkins Health System (JHHS) Corporation’s EPIC-based electronic health record (EHR) data between January 1, 2019, and December 31, 2023, and developed five 1-year cohorts [20]. We identified patients aged ≥18 years, receiving cancer-related care at JHHS, who had 1 or more encounters with the JHHS Cancer Center or a provider specializing in oncology during the study period [20]. This approach enabled us to include a diverse range of patients across the cancer care continuum, from those actively undergoing treatment to cancer survivors. Importantly, our population definition did not depend on the presence of a cancer diagnosis code, allowing us to exclude individuals whose records might reflect such codes for reasons unrelated to current cancer care, such as routine screening tests, historical diagnoses lacking clinical relevance, or instances where patients received care outside of JHHS, which could result in incomplete or missing records and limit the reliability of assessment. Our resulting denominator of 124,974 adult patients had identifiable cancer-related care with JHHS. Of the selected sample, 81,327 (65.1%) had at least one valid 5-digit ZIP code tabulation area (ZCTA) of residence in Maryland during our observation period, and the remaining 43,647 (34.9%) had an out-of-state ZCTA (surrounding states or far away), but we did not exclude any patients based on residency.

### Ethical Considerations

The institutional review board (IRB00424877) of the Johns Hopkins School of Medicine reviewed and approved this study as exempt. The board approved the EHR data extraction for the secondary analysis of deidentified data. The data were deidentified, and the patients did not receive any compensation.

### Variable Definitions

We defined the receipt of telehealth services as an encounter associated with specific Current Procedural Terminology and Healthcare Common Procedure Coding System codes, Current Procedural Terminology modifiers *GT or 95*, Centers for Medicare and Medicaid Services place of service code *02*, or recorded encounter type as *video* or *phone* [21]. A full accounting of our telehealth definition appears in supplemental materials (Table S1 in Multimedia Appendix 1). We further specified whether a telehealth encounter was linked to cancer-related care by flagging cases where it was linked to encounters with an oncology specialist or a department that was part of the JHHS Cancer Center.

We identified several covariates of interest at the patient and ZCTA levels. Overall, we assessed care utilization and comorbidity using the Adjusted Clinical Groups System (ACG) published by Johns Hopkins University [22]. The ACG system identified patient age, sex, presence of any social needs, counts of chronic conditions, medications, total medication gaps, and care utilization for multiple points of care (eg, all-cause hospitalization, emergency department (ED) visits, outpatient encounters identified in each 1-year cohort of the study population). The ACG system used expanded diagnostic clusters (EDCs) from *International Classification of Diseases, Tenth Revision, Clinical Modification* (ICD-10-CM) codes documented in the EHR, including encounter diagnoses, problem lists, and clinical documentation. This validated methodology flagged patients with multiple comorbidities based on relevant diagnoses. Medication counts reflected the number of unique prescriptions recorded in the EHR, including all actively prescribed or renewed medications during the study period. We aggregated binary encounter-level flags for whether a patient required language interpretation services at any point during the observation period.

The ‘comorbidity’ package of the R programming language flagged validated ICD-10-CM codes for cancer and metastatic cancer from the Charlson comorbidity index (CCI) [23]. In addition, the ACG system further subdivided ICD-10-CM codes by EDCs, which generally specifies a body system of neoplasms and other malignancies. However, it is important to note that patients were not required to have a cancer diagnosis confirmed through ICD-10 coding, per the JHHS EHR. We justify this through the observation that the EHR data, compared to administrative claims records, are typically found to be less complete in diagnostic information, including cancers, and the possibility of excluding individuals without ICD-10 coding but with cancer-related care may result in biasing these findings [24].

We obtained counts of cable internet service providers (ISPs) and maximum available download and upload speeds in Gbps from the 2019 report of the Federal Communications Commission’s Connect2Health system and translated them to ZCTA geographies by converting and aggregating values using the ‘zipcodeR’ library of the R programming language [23,25]. We obtained counts of COVID-19 cases between March 2020 and February 2022, as reported by the Maryland Department of Health, through the State of Maryland Open Data Portal [26-28]. We calculated the Area Deprivation Index (ADI) by pulling related variables from the US Census API at the ZCTA level, for the year 2020, using a methodology elaborated on by the original authors [29-32]. Finally, the US Department of Agriculture publishes decennial categories for the category of the rural–urban commuting area (RUCA) at the ZCTA level, but only the 2010 Census release was available at the time of these analyses. The RUCA primary categories were ordinal, ranging from 1 to 10 in order of increasing rurality [21]. We treated the primary categories as continuous values for these analyses to simplify the interpretation of linear effects in predictive modeling. We attached ZCTA-level features of ISPs, maximum available speeds, the cumulative count, and prevalence of COVID-19 cases for the 2 years following the start of the pandemic, national-ranked ADI, and the primary RUCA to match patient records.

### Statistical Tests

We used a combination of statistical modeling and null hypothesis tests to assess patterns of telehealth utilization and its association with health care outcomes (ie, hospitalization, ED visitation, and elevated resource utilization). Thus, we performed multiple comparisons between those receiving or not having received cancer-related telehealth services between 2019 and 2023 and evaluated for significant effects at any point during the observation period using *χ*^2^ tests of independence. We assessed both demographic and clinical characteristics, including comorbidities, care utilization, and cancer type (defined by the EDC malignancy groups). We set the alpha level for patient-level significance testing at .001 to reduce the frequency of spurious findings due to sample size.

We cross-tabulated the geospatial characteristics by quartile of telehealth utilization at the ZCTA level, allowing for missingness wherever our sample did not have a valid ZCTA of residence listed. We suppressed areas with fewer than 11 patients for privacy. The quartiles contained an equal number of ZCTAs of residence and were categorized from the Maryland ZCTA containing the lowest proportion of residents observed to have cancer-related telehealth services at JHHS (quartile 1) to the highest number (quartile 4). The out-of-state ZCTA (surrounding states or far away) was all put together in a separate group. This category also included patients missing the ZCTA information. Visual inspection of trends permitted a general assessment of whether geospatial characteristics were associated with telehealth utilization in aggregate and were confirmed by Spearman ρ for significance testing and using only valid geographies. We set the α level for ZCTA-level hypothesis tests at .05.

We used patient-year aggregated observations in the modeling of concurrent year cancer-related telehealth utilization and for assessing its association with health care outcomes of hospitalization, ED visitation, and elevated resource utilization (ie, ACG system Resource Utilization Band [RUB] of 4 or 5 categories). RUB represents expected future utilization based on current morbidities. It is presented in the following categories: (1) healthy users, (2) low resource utilization, (3) moderate resource utilization, (4) high resource utilization, and (5) very high resource utilization [22].

We fitted a generalized estimating equation model with an exchangeable correlation structure for patient identifiers, given the repeated nature of observations across years of data. We evaluated the model for performance and variable effects as part of an analysis to assess the factors associated with receiving cancer-related telehealth services. The model consisted of patient age, sex, need for interpreter services, presence of any social need, counts of chronic conditions, medications, total medication gaps, outpatient visits count, a binary flag for each EDC associated with malignancies or neoplasms, and all ZCTA-level geospatial features. The model would help to assess the association of different types of cancer (defined by the EDC variables) with the outcome of interest. We fitted another model consisting of the same features as the first model, with a CCI-defined cancer diagnosis instead of the binary flag for the EDC groups. This model would help to assess how the high-level information regarding the severity of cancer (defined through the CCI variable) would impact the use of cancer-related telehealth services.

We performed a similar set of analyses to assess the telehealth association with health care outcomes (ie, hospitalization, ED visitation, and elevated resource utilization). In addition to the factors listed earlier, this set of models included a flag for whether the patient received cancer-related telehealth services.

We converted the effects of coefficients to odds ratios with 95% CIs to detect significant effects. We completed all models and plots using the R programming language (version 4.0.2). We compared the model performances on area under the receiver operating characteristic curve and area under the precision-recall curve, a measure of success of prediction when the classes are very imbalanced, along with point-estimated positive predictive value (PPV) and sensitivity.

## Results

### Patient Characteristics

Table 1 presents the high-level characteristics of the study cohort. The proportion of patients not receiving and receiving cancer-related telehealth services during the observation period was split 69.4% to 30.6% (86,732 vs 38,242 patients). Patients receiving cancer-related telehealth were significantly older on average (19,942, 52.2% vs 42,209, 48.7% for patients aged ≥65 years, *P*<.001) and more often male (17,365, 45.4% vs 34,839, 40.2%). They were significantly more likely to identify as White race (27,071, 70.8% vs 56,122, 64.7%), have 3 or more chronic conditions (23,503, 61.5% vs 33,000, 38.0%), take 3 or more medications (37,104, 97.0% vs 73,623, 84.9%), have hospitalizations (16,096, 42.1% vs 21,834, 25.2%), and ED visits (8644, 22.6% vs 15,590, 18.0%). Patients with cancer-related telehealth care were also much more likely to be identified as having high resource utilization (22,695, 59.3% vs 30,464, 35.1%; Table S2 in Multimedia Appendix 1 presents patient characteristics of the study cohort by quartiles of telehealth utilization at the ZCTA level).

### Patterns of Telehealth Utilization

Table 1 presents the high-level characteristics of the study cohort. The proportion of patients not receiving and receiving cancer-related telehealth services during the observation period was split 69.4% to 30.6% (86,732 vs 38,242 patients). Patients receiving cancer-related telehealth were significantly older on average (19,942, 52.2% vs 42,209, 48.7% for patients aged ≥65 years, *P*<.001) and more often male (17,365, 45.4% vs 34,839, 40.2%). They were significantly more likely to identify as Caucasian race (27,071, 70.8% vs 56,122, 64.7%), have 3 or more chronic conditions (23,503, 61.5% vs 33,000, 38.0%), take 3 or more medications (37,104, 97.0% vs 73,623, 84.9%), have hospitalizations (16,096, 42.1% vs 21,834, 25.2%), and ED visits (8644, 22.6% vs 15,590, 18.0%). Patients with cancer-related telehealth care were also much more likely to be identified as having high resource utilization (22,695, 59.3% vs 30,464, 35.1%; Table S2 in Multimedia Appendix 1 presents patient characteristics of the study cohort by quartiles of telehealth utilization at the ZCTA level).

**Table 1.** Patient characteristics for the cohort receiving cancer care from the Johns Hopkins health system between 2019 and 2023: those with and without cancer-related telehealth.

Feature	No cancer-related telehealth, n (%)	Any cancer-related Telehealth^a^, n (%)	Total, n (%)	*χ*^2^ test^b^ (*df*)
Total patients with cancer	86,732 (100)	38,242 (100)	124,974 (100)	
Age (y)				
18‐44	17,090 (19.7)	5252 (13.7)	22,342 (17.9)	728.0 (3)
45‐64	27,433 (31.6)	13,048 (34.1)	40,481 (32.4)	
65‐79	30,517 (35.2)	15,100 (39.5)	45,617 (36.5)	
≥80	11,692 (13.5)	4842 (12.7)	16,534 (13.2)	
Sex				
Female	51,822 (59.7)	20,868 (54.6)	72,690 (58.2)	296.3 (1)
Male	34,839 (40.2)	17,365 (45.4)	54,204 (43.4)	
Race				
African American	16,511 (19)	7031 (18.4)	23,542 (18.8)	938.8 (3)
Asian	4108 (4.7)	1801 (4.7)	5909 (4.7)	
White	56,122 (64.7)	27,071 (70.8)	83,193 (66.6)	
Other/unknown	9991 (11.5)	2339 (6.1)	12,330 (9.9)	
English proficiency				
Need for an interpreter	2537 (2.9)	702 (1.8)	3239 (2.6)	124.3 (1)
Social needs				
Have at least one social need	9455 (10.9)	6340 (16.6)	15,795 (12.6)	744.2 (1)
Clinical characteristics^c^				
Patients with ≥3 chronic conditions	33,000 (38.0)	23,503 (61.5)	56,503 (45.2)	5870.9 (1)
Patients with ≥3 medications	73,623 (84.9)	37,104 (97.0)	110,727 (88.6)	3870.5 (1)
Health care utilization				
Patients with ≥1 hospitalization	21,834 (25.2)	16,096 (42.1)	37,930 (30.4)	3591.7 (1)
Patients with ≥1 ED visits	15,590 (18.0)	8644 (22.6)	24,234 (19.4)	363.5 (1)
Resource Utilization Band 4‐5	30,464 (35.1)	22,695 (59.3)	53,159 (42.5)	6369.1 (1)
Type of cancer				
Neoplasms of the skin	3108 (3.6)	2273 (5.9)	5381 (4.3)	358.3 (1)
Low-impact neoplasms^d^	10,994 (12.7)	10,805 (28.3)	21,799 (17.4)	4471.7 (1)
High-impact neoplasms^d^	12,356 (14.2)	13,877 (36.3)	26,233 (21)	7773 (1)
Neoplasm, breast	10,596 (12.2)	6245 (16.3)	16,841 (13.5)	384.8 (1)
Neoplasm, cervix	2316 (2.7)	1255 (3.3)	3571 (2.9)	35.5 (1)
Neoplasm, ovary	1397 (1.6)	750 (2)	2147 (1.7)	19.1 (1)
Neoplasm, esophagus	570 (0.7)	567 (1.5)	1137 (0.9)	199.7 (1)
Neoplasm, kidney	928 (1.1)	816 (2.1)	1744 (1.4)	217.5 (1)
Neoplasm, liver	1528 (1.8)	1558 (4.1)	3086 (2.5)	588.3 (1)
Neoplasm, lung	3157 (3.6)	3143 (8.2)	6300 (5)	1161.4 (1)
Neoplasm, lymphoma	3593 (4.1)	2517 (6.6)	6110 (4.9)	339 (1)
Neoplasm, colorectal	2755 (3.2)	2386 (6.2)	5141 (4.1)	630.4 (1)
Neoplasm, pancreas	2463 (2.8)	2397 (6.3)	4860 (3.9)	833.6 (1)
Neoplasm, prostate	5741 (6.6)	4237 (11.1)	9978 (8)	718.1 (1)
Neoplasm, stomach	426 (0.5)	437 (1.1)	863 (0.7)	163.3 (1)
Neoplasm, acute leukemia	1992 (2.3)	1464 (3.8)	3456 (2.8)	230.9 (1)
Neoplasm, bladder	1173 (1.4)	932 (2.4)	2105 (1.7)	187.9 (1)

a Patients could receive cancer-related telehealth services at any point during the observation period.

b Statistically significant with *P*<.001.

c These clinical measures are derived from the Johns Hopkins Adjusted Clinical Groups System Version 12.0. The Resource Utilization Band represents expected future utilization based on current morbidities. It is presented in the following categories: (1) healthy users, (2) low resource utilization, (3) moderate resource utilization, (4) high resource utilization, and (5) very high resource utilization.

d Low- and high-impact neoplasm groups do not capture severity for all cancers. Rather, they include cancers that are not specified in other expanded diagnostic cluster groups. For example, the expanded diagnostic cluster groups do not include head and neck cancer malignancies, so cancers for those sites would be mapped to low- and high-impact neoplasm groups.

Table 1 also illustrates malignancies by EDC with respect to telehealth utilization. Our significance testing identified a greater proportion of telehealth patients for each EDC group, with the smallest significant finding for ovarian cancers (*χ*^2^=19.1, *P*<.001). We observed the highest differences in rate for low-impact neoplasms among those with no telehealth versus telehealth use (10,994, 12.7% vs 10,805, 28.3%, respectively), high-impact neoplasms (12,356, 14.2% vs 13,877, 36.3%, respectively), lung cancers (3157, 3.6% vs 3143, 8.2%, respectively), and pancreatic cancer (2463, 2.8% vs 2397, 6.3%, respectively), and each was associated with more than twice the rate among those with cancer-related telehealth encounters.

We broke the Maryland ZCTAs into quartiles for the proportion of residents observed to have cancer-related telehealth services at JHHS. When split into quartiles, the ZCTA covered by the JHHS in this analysis included 325 (69.4%) of the 468 Maryland ZCTA codes, mostly reflective of the central and capital regions of the state. Patients arrived at JHHS points of care from everywhere in the state (Figure S1 in Multimedia Appendix 1). The number of patients with any telehealth use increased from 17,687 (56.5%) in quartile 1 to 16,244 (58.9%) in quartile 2, 9924 (59.4%) in quartile 3, and 3527 (61.4%) in quartile 4. The number of patients with any cancer-related telehealth use increased from 8352 (26.7%) in quartile 1 to 8788 (31.9%) in quartile 2, 6126 (36.7%) in quartile 3, and 2545 (44.3%) in quartile 4. We put together the out-of-state ZCTA (surrounding states or far away) in a separate group. This category also included patients missing the ZCTA information. There were a total of 110 ZCTA codes in this category. Of 543 patients in this category, 331 (61.0%) received any telehealth services, and 237 (43.6%) received any cancer-related telehealth services. Most notably, patients in this category resided in Washington, DC, a heavily populated metropolitan area.

Table 2 presents the characteristics of the patient’s place of residence by ZCTA quartiles. Some of the most notable findings were that with increasing telehealth utilization in the aggregate, the primary RUCA code shifted from core metropolitan areas (code 1) to metropolitan noncore (codes 2‐3) and micropolitan, small town, or rural (codes 4‐10).

**Table 2.** Characteristics of the patient’s place of residence at 5-digit ZIP code tabulation area by quartiles of telehealth utilization.^a^

Characteristics of patient’s place of residence	Maryland ZCTA^b^				Other ZCTA
	Quartile 1	Quartile 2	Quartile 3	Quartile 4	
Total population, 2020	1,918,616	1,773,007	1,587,433	624,630	81,304
ZCTA codes (n)	82	81	81	81	110
Patients in the sample (n)	31,295	27,580	16,711	5741	543
Socioeconomic characteristics of area, mean (SD)					
Average National Rank ADI^c^	21.1 (21.3)	15.7 (14.3)	15.8 (10.5)	17.8 (14.8)	34.4 (29.3)
Primary RUCA^d^ of area, n (%)					
Metropolitan—core	63 (76.8)	59 (72.8)	51 (63)	28 (34.6)	21 (19.1)
Metropolitan—noncore	17 (20.7)	20 (24.7)	22 (27.2)	35 (43.2)	62 (56.4)
Micropolitan	0 (0)	1 (1.2)	4 (4.9)	7 (8.6)	13 (11.8)
Small town, rural	2 (2.4)	1 (1.2)	4 (4.9)	11 (13.6)	14 (12.7)
COVID-19 prevalence in area, mean (SD)					
Cumulative per 100,000 residences (through February 2022)	17,097.2 (5169.2)	15,404.1 (3439.4)	16,140.0 (3730.4)	15,042.6 (4112.1)	17,448.5 (11,077.7)
Internet connectivity in area, mean (SD)					
Count of cable ISPs^e^ per ZCTA	2.5 (0.8)	2.5 (0.8)	2.4 (0.8)	2.3 (0.9)	1.7 (0.7)
Max cable download speed in Mbps	988.7 (9.4)	983.7 (82.8)	991.0 (6.0)	956.4 (162.1)	892.0 (244.4)
Max cable upload speed in Mbps	828.4 (226.8)	834.4 (249.6)	752.0 (335.1)	529.3 (441.8)	364.0 (425.7)

a Areas where the sample count is <11 patients are removed from the sample. The quartiles contain an equal number of ZIP codes and are categorized from the ZIP codes in Maryland containing the lowest proportion of residents observed to have cancer-related telehealth services at Johns Hopkins Health System (Q1) to the highest number (Q4). The out-of-state ZIP codes (surrounding states or far away) are all put together in a separate group. This category also includes patients missing the ZIP code information.

b ZCTA: 5-digit ZIP Code Tabulation Area.

c ADI: Area Deprivation Index (the higher the number, the greater socioeconomic challenges).

d RUCA: Rural–Urban Commuting Area.

e ISP: internet service provider.

The RUCA code was positively and significantly associated with the percentage of the sample using cancer-related telehealth services (ρ=0.36; *P*<.05). Moreover, the average maximum of available download and upload speeds dropped as well (ρ=−0.22; *P*<.05; and ρ=−0.34, *P*<.05, respectively), signifying an inverse relationship between telehealth utilization and internet speeds, but not the number of cable ISPs (ρ=−0.09). Telehealth utilization had no significant aggregate association with nationally ranked ADI (ρ=0.01) but did for COVID-19 prevalence (ρ=0.36, *P*<.05; Figure S2 in Multimedia Appendix 1 provides detailed information on reported correlations).

Table 3 presents odds ratios and 95% CIs for the model containing the EDC-derived markers assessing the concurrent-year cancer-related telehealth utilization. After controlling for other factors, cancer-related telehealth usage was high among males (1.062, 1.032:1.093), those with chronic conditions (1.034, 1.03:1.039), higher number of medications (1.036, 1.035:1.037), and outpatient care utilization (1.026, 1.026:1.027). Moreover, it was higher among those living in more rural areas (ie, higher primary RUCA, 1.117, 1.1:1.134), areas with higher COVID-19 prevalence (1.001, 0.999:1.004), and higher maximum cable internet download speed (1.717, 1.292:2.282). In assessing the cancer type and severity, the model found the strongest significant associations between greater cancer-related telehealth usage with malignant neoplasms of the prostate and pancreas (1.953, 1.87:2.04 and 1.82, 1.731:1.913, respectively), as well as low- and high-impact malignant neoplasms (1.712, 1.662:1.764 and 1.701, 1.654:1.749, respectively).

**Table 3.** Assessing the concurrent year cancer-related telehealth utilization for patients receiving cancer care at Johns Hopkins Health System: adjusted odds ratios associated with key patient or geographic factors.^a^

Key factor	Cancer-related telehealth utilization
Demographic characteristics	
Age (y)	0.995 (0.995:0.996)
Sex–male (ref: female)	1.062 (1.032:1.093)
English proficiency (need for an interpreter)	0.601 (0.55:0.657)
Any social needs	0.851 (0.81:0.893)
Clinical characteristics^b^	
Chronic conditions count	1.034 (1.03:1.039)
Medication count	1.036 (1.035:1.037)
Total medication gaps	0.931 (0.92:0.942)
Outpatient visits count	1.026 (1.026:1.027)
Expanded diagnostic cluster associated with malignancies or neoplasms	
MAL01: malignant neoplasms of the skin	1.01 (0.957:1.066)
MAL02: low-impact malignant neoplasms^c^	1.712 (1.662:1.764)
MAL03: high-impact malignant neoplasms^c^	1.701 (1.654:1.749)
MAL04: malignant neoplasms, breast	1.576 (1.522:1.631)
MAL05: malignant neoplasms, cervix, uterus	0.981 (0.916:1.05)
MAL06: malignant neoplasms, ovary	0.929 (0.851:1.015)
MAL07: malignant neoplasms, esophagus	1.447 (1.3:1.61)
MAL08: malignant neoplasms, kidney	1.355 (1.24:1.482)
MAL09: malignant neoplasms, liver, and biliary tract	1.359 (1.276:1.447)
MAL10: malignant neoplasms, lung	1.656 (1.579:1.736)
MAL11: malignant neoplasms, lymphomas	1.22 (1.156:1.288)
MAL12: malignant neoplasms, colorectal	1.607 (1.528:1.69)
MAL13: malignant neoplasms, pancreas	1.82 (1.731:1.913)
MAL14: malignant neoplasms, prostate	1.953 (1.87:2.04)
MAL15: malignant neoplasms, stomach	1.504 (1.334:1.694)
MAL16: acute leukemia	0.75 (0.688:0.817)
MAL18: malignant neoplasms, bladder	1.065 (0.982:1.155)
Geospatial characteristics	
Year	1.202 (1.196:1.209)
National Rank ADI^d^	0.991 (0.99:0.991)
Primary RUCA^e^	1.117 (1.1:1.134)
COVID-19 prevalence^f^	1.001 (0.999:1.004)
Count of cable ISPs^g^ per ZCTA^h^	0.999 (0.98:1.019)
Max cable download speed in Mbps	1.717 (1.292:2.282)
Max cable upload speed in Mbps	0.662 (0.618:0.71)

a The odds ratio (95% CIs) is presented for each variable included in the model and for the concurrent year outcome. The model includes a binary flag for each expanded diagnostic cluster (EDC) associated with malignancies or neoplasms to assess the association of different types of cancer (defined by the EDC variables) with the outcome of interest.

b These clinical measures are derived from the Johns Hopkins Adjusted Clinical Group System version 12.0.

c Low- and high-impact neoplasm groups do not capture severity for all cancers. Rather, they include cancers that are not specified in other EDC groups. For example, the EDC groups do not include head and neck cancer malignancies, so cancers for those sites would be mapped to low and high-impact neoplasm groups.

d ADI: Area Deprivation Index.

e The Rural-Urban Commuting Area (RUCA) primary categories are ordinal, ranging from 1 to 10 in order of increasing rurality. We treat the primary categories as continuous values for these analyses to simplify the interpretation of linear effects in predictive modeling.

f Cumulative prevalence per 100,000 residences through February 2022.

g ISP: internet service provider.

h ZCTA: 5-digit ZIP Code Tabulation Area.

### Association of Telehealth With Health Care Outcomes

Table 4 presents odds ratios and 95% CIs for the model containing the EDC-derived markers assessing the concurrent-year health care outcomes. Concurrent year receipt of cancer-related telehealth services was significantly associated with reductions in odds of ED utilization (0.913, 0.882:0.946) and hospitalization (0.78, 0.75:0.811). Despite these findings, overall concurrent year resource utilization was still high among those receiving cancer-related telehealth (1.148, 1.113:1.185), even after controlling for chronic conditions (2.224, 2.205:2.242), medications (1.102, 1.1:1.104), outpatient care utilization (1.022, 1.021:1.023), and social needs and need for an interpreter (1.811, 1.714:1.914 and 1.526, 1.405:1.657, respectively). In assessing the cancer type and severity, the model found a significant association between high-impact malignancies with greater hospitalization (2.033, 1.954‐2.116), but not ED utilization (0.908, 0.873:0.944) and elevated RUB (0.915, 0.884‐0.947). Several individual EDC malignancies had lower ED and inpatient care utilization but higher RUB. These included skin neoplasms (1.132, 1.062‐1.207), breast cancer (1.274, 1.220‐1.334), ovarian cancer (1.249, 1.124‐1.388), pancreatic cancer (1.070, 1.001‐1.144), and leukemia (1.404, 1.300‐1.517).

**Table 4.** Assessing the concurrent year health care outcomes for patients receiving cancer care at Johns Hopkins Health System: adjusted odds ratios associated with key patient or geographic factors.^a^

Key factor	ED^b^ visit^c^	Hospitalization^c^	Expected future utilization of higher cost^c^
Telehealth utilization			
Any cancer-related telehealth during the year	0.913 (0.882:0.946)	0.78 (0.75:0.811)	1.148 (1.113:1.185)
Demographic characteristics			
Age (y)	0.994 (0.993:0.995)	0.987 (0.986:0.989)	0.994 (0.993:0.995)
Sex–male (ref: female)	0.866 (0.835:0.898)	1.334 (1.281:1.388)	1.282 (1.238:1.326)
English proficiency (need for an interpreter)	1.568 (1.435:1.714)	2.534 (2.312:2.778)	1.526 (1.405:1.657)
Any social needs	1.813 (1.727:1.904)	1.477 (1.401:1.556)	1.811 (1.714:1.914)
Clinical characteristics^c^			
Chronic conditions count	1.061 (1.056:1.067)	1.259 (1.251:1.266)	2.224 (2.205:2.242)
Medication count	1.054 (1.053:1.056)	1.22 (1.218:1.222)	1.102 (1.1:1.104)
Total medication gaps	1.05 (1.038:1.063)	0.896 (0.885:0.908)	0.931 (0.919:0.944)
Outpatient visits count	1.009 (1.008:1.01)	0.989 (0.989:0.99)	1.022 (1.021:1.023)
Expanded diagnostic cluster associated with malignancies or neoplasms			
MAL01: malignant neoplasms of the skin	0.781 (0.726:0.841)	0.558 (0.515:0.606)	1.132 (1.062:1.207)
MAL02: low-impact malignant neoplasms^d^	0.854 (0.821:0.889)	1.099 (1.054:1.147)	1.375 (1.328:1.424)
MAL03: high-impact malignant neoplasms^d^	0.908 (0.873:0.944)	2.033 (1.954:2.116)	0.915 (0.884:0.947)
MAL04: malignant neoplasms, breast	0.858 (0.82:0.898)	0.49 (0.461:0.52)	1.274 (1.22:1.33)
MAL05: malignant neoplasms, cervix, uterus	0.964 (0.89:1.044)	1.095 (0.997:1.202)	1.228 (1.129:1.334)
MAL06: malignant neoplasms, ovary	0.932 (0.84:1.035)	0.872 (0.774:0.982)	1.249 (1.124:1.388)
MAL07: malignant neoplasms, esophagus	0.621 (0.529:0.73)	0.922 (0.79:1.077)	0.985 (0.849:1.142)
MAL08: malignant neoplasms, kidney	1.014 (0.903:1.14)	1.217 (1.081:1.369)	1.144 (1.031:1.27)
MAL09: malignant neoplasms, liver, and biliary tract	0.917 (0.84:1.001)	1.541 (1.412:1.683)	1.548 (1.428:1.677)
MAL10: malignant neoplasms, lung	1.001 (0.942:1.064)	1.354 (1.267:1.447)	1.424 (1.344:1.508)
MAL11: malignant neoplasms, lymphomas	0.802 (0.745:0.862)	1.048 (0.974:1.128)	1.337 (1.261:1.418)
MAL12: malignant neoplasms, colorectal	0.898 (0.836:0.965)	1.332 (1.238:1.432)	1.373 (1.29:1.46)
MAL13: malignant neoplasms, pancreas	0.552 (0.504:0.604)	0.741 (0.683:0.805)	1.07 (1.001:1.144)
MAL14: malignant neoplasms, prostate	1.034 (0.974:1.099)	0.643 (0.599:0.689)	0.912 (0.865:0.962)
MAL15: malignant neoplasms, stomach	0.945 (0.804:1.111)	1.357 (1.146:1.606)	1.135 (0.968:1.332)
MAL16: acute leukemia	0.357 (0.315:0.405)	0.971 (0.882:1.068)	1.404 (1.3:1.517)
MAL18: malignant neoplasms, bladder	1.103 (1.001:1.216)	0.788 (0.705:0.881)	0.93 (0.846:1.023)
Geospatial characteristics			
Year	0.969 (0.961:0.977)	0.906 (0.898:0.914)	1.035 (1.027:1.043)
National Rank ADI^e^	1.008 (1.001:1.014)	1.005 (1.003:1.006)	1.005 (1.004:1.006)
Primary RUCA^f^	0.414 (0.361:0.476)	0.897 (0.869:0.926)	0.927 (0.905:0.949)
COVID-19 prevalence^g^	0.725 (0.38:1.385)	0.988 (0.981:0.995)	0.999 (0.996:1.002)
Count of cable ISPs^h^ per ZCTA^i^	1.226 (1.196:1.256)	1.078 (1.047:1.11)	1.007 (0.983:1.031)
Max cable download speed in Mbps	1.878 (0.997:3.536)	0.855 (0.543:1.345)	1.138 (0.783:1.654)
Max cable upload speed in Mbps	1.098 (0.965:1.248)	0.86 (0.773:0.955)	1.04 (0.951:1.138)

a The odds ratio (95% CIs) is presented for each variable included in the model and for each concurrent year outcome. The model includes a binary flag for each expanded diagnostic cluster (EDC) associated with malignancies or neoplasms to assess the association of different types of cancer (defined by the EDC variables) with the outcome of interest.

b ED: emergency department.

c These clinical measures are derived from the Johns Hopkins Adjusted Clinical Group (ACG) System version 12.0. Expected future utilization of higher cost presents the Resource Utilization Band from the ACG system in the following categories: (1) healthy users, (2) low resource utilization, (3) moderate resource utilization, (4) high resource utilization, and (5) very high resource utilization.

d Low- and high-impact neoplasm groups do not capture severity for all cancers. Rather, they include cancers that are not specified in other EDC groups. For example, the EDC groups do not include head and neck cancer malignancies, so cancers for those sites would be mapped to low- and high-impact neoplasm groups.

e ADI: Area Deprivation Index.

f The Rural-Urban Commuting Area (RUCA) primary categories are ordinal, ranging from 1 to 10 in order of increasing rurality. We treat the primary categories as continuous values for these analyses to simplify the interpretation of linear effects in predictive modeling.

g Cumulative prevalence per 100,000 residences through February 2022.

h ISP: Internet Service Provider,

i ZCTA: 5-digit ZIP Code Tabulation Area.

Table 5 summarizes the prediction metrics of cancer-related telehealth utilization and different health care utilization outcomes. Some of the notable findings were high area under the receiver operating characteristic curves among all models (0.802-0.968). However, the models had a higher PPV (0.375-0.826) than sensitivity (0.049-0.721; Tables S3-S5 in Multimedia Appendix 1 provides details of models including the CCI-defined cancer diagnosis).

**Table 5.** Model performance metrics for assessing the concurrent year cancer-related telehealth utilization and health care utilization outcomes for patients receiving cancer care between 2019 and 2023.

Model performance metrics	Values
Area under the receiver operating characteristic curve	
Cancer-related telehealth utilization^a^	0.802
ED^b^ visit^c^	0.818
Hospitalization^c^	0.960
Elevated (4-5) Resource Utilization Band^c^^d^	0.968
Area under the precision–recall curve	
Cancer-related telehealth utilization^a^	0.345
ED visit^c^	0.219
Hospitalization^c^	0.762
Elevated (4-5) Resource Utilization Band^c^^d^	0.873
Positive predictive value	
Cancer-related telehealth utilization^a^	0.562
ED visit^c^	0.375
Hospitalization^c^	0.747
Elevated (4-5) Resource Utilization Band^c^^d^	0.826
Sensitivity	
Cancer-related telehealth utilization^a^	0.142
ED visit^c^	0.049
Hospitalization^c^	0.610
Elevated (4-5) Resource Utilization Band^c^^d^	0.721

a The model consists of patient age, sex, need for interpreter services, presence of any social need, counts of chronic conditions, medications, total medication gaps, outpatient visits count, a binary flag for each associated with malignancies or neoplasms, and all 5-digit ZIP Code Tabulation Area–level geospatial features.

b ED: emergency department.

c The model consists of patient age, sex, need for interpreter services, presence of any social need, counts of chronic conditions, medications, total medication gaps, outpatient visits count, a binary flag for each expanded diagnostic cluster associated with malignancies or neoplasms, a flag for whether the patient received cancer-related telehealth services and all 5-digit ZIP Code Tabulation Area–level geospatial features.

d Resource Utilization Band is derived from the Johns Hopkins Adjusted Clinical Group System version 12.0. and represents expected future utilization based on current morbidities. It is presented in the following categories: (1) healthy users, (2) low resource utilization, (3) moderate resource utilization, (4) high resource utilization, and (5) very high resource utilization.

## Discussion

### General Findings

In this study, we examined the patterns of telehealth utilization across the cancer care continuum and the factors associated with the receipt of telehealth. Our findings showed that in our large academic medical center, the receipt of cancer-related telehealth care varied by sociodemographic characteristics, type of cancer, and health care resource utilization level among our patient cohort.

Telehealth seems particularly well-suited for cancer care delivery. During cancer care, patients and caregivers may face unique challenges such as the need for more frequent visits than required for other health conditions [33]. Moreover, challenges with transportation, high cost of care, and restricted access to providers can serve to limit utilization of in-person medical, psychological, and supportive care among patients with cancer, especially those with complex comorbidities or social needs [34-36]. Telehealth can reduce the burden of traveling to multiple visits within a short period or traveling when a patient with cancer is experiencing side effects from treatment.

Furthermore, in the oncology care context, telehealth has been shown to reduce wait times, expedite cancer diagnosis and treatment (compared to in-person visits), improve symptom management and comfort, reduce health care utilization, and increase patient satisfaction [37-39]. The implementation of telehealth strategies has also helped to address gaps in cancer-related care associated with geographic access that could otherwise result in differences in the cost of care and cancer-related outcomes [40]. Thus, across the cancer care continuum, telehealth has been an important mode of care delivery, especially in ambulatory care settings and in locations where in-person access is limited due to social conditions or economic challenges [41-46].

### Comparison With Other Studies

Our adjusted analyses found that patients who received cancer-related telehealth were significantly more likely to be younger and male. Current evidence shows that the receipt of telehealth (overall and among cancer survivors) is significantly lower in patients who are members of racial minorities, older, rural-dwelling, and socioeconomically challenged, compared to those who are Whites, younger, urban-dwelling, and wealthier [7,18]. In addition, national population-based studies report greater receipt of telehealth among younger individuals and females [7,8,47]. This divergence in gender use rates captured by our study may reflect differences in the reasons for cancer-related telehealth use versus telehealth that is unrelated to cancer care (eg, a very large percentage of national telehealth being used for behavioral health).

Our adjusted analyses found that patients who received cancer-related telehealth were significantly more likely to be younger and male. Current evidence shows that the receipt of telehealth (overall and among cancer survivors) is significantly lower in patients who are members of racial minorities, older, rural-dwelling, and socioeconomically challenged, compared to those who are Caucasians, younger, urban-dwelling, and wealthier [7,18]. In addition, national population-based studies report greater receipt of telehealth among younger individuals and females [7,8,47]. This divergence in gender use rates captured by our study may reflect differences in the reasons for cancer-related telehealth use versus telehealth that is unrelated to cancer care (eg, a very large percentage of national telehealth being used for behavioral health).

Also in our study, patients with at least one social need received fewer cancer-related telehealth services compared to those with no social needs. This finding may indicate that socioeconomic challenges can impact patients’ access to any care, including that provided through telehealth. Other studies have identified sociodemographic differences associated with age, sex, race/ethnicity, and health insurance type in receipt of video-based telehealth (compared with phone-only telehealth) [7,48-50]. Research that identifies potential contributors to different patterns of telehealth utilization in cancer care and the association between cancer-related telehealth utilization and health care outcomes can inform cancer management strategies [8]. Understanding correlates of receipt of telehealth among individuals diagnosed with cancer who are receiving care across the cancer care continuum and in different clinical settings (eg, at a cancer center or a primary care clinic) can help identify gaps in care.

In our study, patients with a higher number of chronic conditions, medications, and outpatient visits were more likely to receive cancer-related telehealth. This is concordant with previous general population research that found greater receipt of telehealth among individuals with more comorbidities [8]. These findings may indicate a preference (or need) for telehealth among patients with more comorbidities and complex conditions, given the utility of telehealth in reducing some access barriers. Also, patients with high-impact neoplasms, prostate, and pancreatic cancer had higher rates of cancer-related telehealth use than other types of cancer. These findings further suggest that telehealth may be particularly well-suited for patients with certain types of more serious cancer care needs. However, the findings that patients with low-impact neoplasms were also more likely to receive cancer-related telehealth may indicate that patients with less complex health needs may seek types of care that are more suitable for telecare or that these patients prefer fewer in-person interactions with health care. Additional research to understand patient preferences for telehealth among people with cancer is needed.

Analyses examining quartiles of cancer-related telehealth use and the correlation matrix for geospatial characteristics revealed informative findings. Consistent with prior research [7,18], our study found a positive association between cancer-related telehealth use and rural areas of residence, with the percentage of the sample using telehealth increasing with the rurality of where they lived. This finding was confirmed in the adjusted analyses. In addition, the adjusted analyses showed higher cancer-related telehealth use among those living in areas with higher COVID-19 prevalence and higher access to the internet (as measured by maximum available internet download speed). These findings may indicate the effectiveness of telehealth in overcoming geographic access challenges (eg, associated with distance and limited transportation) among patients with cancer living in rural areas [51] or when public health emergencies such as a pandemic emerge. Strategies that maximize, or otherwise support, telehealth availability in rural areas are warranted.

Our findings suggested that after adjustment, concurrent receipt of cancer-related telehealth was associated with lower odds of ED utilization and hospitalization. This is concordant with several other studies (both overall and within the cancer context) that likewise report associations between telehealth and reductions in ED utilization or hospitalization [52-55]. A scoping review of telehealth during the COVID-19 pandemic concluded that telehealth could reduce hospital admission rates [56]. In a surgical oncology study, postoperative receipt of telehealth reduced ED visits and 30-day readmissions [55]. It appears that the use of timely digital care decreases ED visits and hospital admissions, which can reduce cancer care costs, improve quality of care, and potentially protect patients with cancer, who are susceptible to infections. A population-based study found that among patients with cancer, ED visits increased from an estimated 3.7 million in 2012 to 6.2 million in 2019, with more than half of these visits identified as potentially preventable [57]. Combined with the overall effectiveness of telehealth in reducing the time and travel costs of patients with cancer, continued use of telehealth in cancer care may have a significant impact on cancer care costs and cancer-related financial toxicity [58].

Our findings related to hospitalization and severity/disease complexity were somewhat paradoxical. High-impact malignancies were associated with greater rates of hospitalization, but patients with cancer with higher overall comorbidities (across all conditions as measured by our ACG-RUB measure) were not more likely to be hospitalized. This finding may be due to higher in-hospital death rates among high-impact malignancies. Also, certain EDCs representing ICD-10 codes falling within selected malignancy categories (eg, skin neoplasms, breast cancer, ovarian cancer, pancreatic cancer, and leukemia) did not consistently reach levels of significance for each outcome, but several were significant and had the same pattern as cancer-related telehealth: lower ED and inpatient care utilization and higher elevated RUB. Lower ED visits and hospitalizations may reflect high survivability. However, in the case of pancreatic cancer or leukemia, it may reflect a higher use of palliative care. Further research in this and other settings on this topic is warranted.

In terms of the model performance, the inclusion of individual EDC diagnosis clusters specifying malignancy type in the place of CCI-defined cancer diagnosis appeared to have substantially improved both PPV and sensitivity for cancer-related telehealth utilization and hospitalization and PPV for ED visitation. In addition, area under the precision-recall curve was improved with this approach, suggesting better precision-recall across much of the response distribution (Tables S3-S5 in Multimedia Appendix 1 provide details of models, including the CCI-defined cancer diagnosis).

### Study Limitations

Our analysis relied on a binary measure of telehealth exposure (received or not) due to limitations in the EHR dataset, which did not uniformly capture telehealth modality (eg, phone vs video), frequency, or clinical purpose. While these granular data could deepen insights into how telehealth encounters affect outcomes such as ED visits and hospitalizations, they were unavailable for inclusion. Future research should aim to incorporate such measures to optimize telehealth delivery and better inform tailored interventions.

In addition, the endpoints used to assess telehealth’s association with health care outcomes were limited. Other clinically relevant measures, such as laboratory test use and medication adjustments, or hybrid models integrating telehealth and in-person care, could offer more comprehensive insights into its effects on outcomes.

The reliance on EHR data may have affected the identification of telehealth services due to potential coding inaccuracies. Furthermore, this study focused on patients within a single large academic health system, which may not fully represent broader cancer populations. JHHS’s specialized programs, advanced technological infrastructure, and patient demographics—such as higher proportions of insured or resource-accessible patients—limit external generalizability. Patients in other settings, such as rural clinics or community hospitals, may have distinct experiences and patterns of telehealth use. Future research across diverse health care systems is needed to ensure findings are applicable to more varied populations, particularly in rural and underserved areas.

The COVID-19 pandemic accelerated telehealth adoption, addressing barriers such as long travel distances, workforce shortages, and limited health care facilities. However, sustaining these access levels beyond the pandemic is uncertain due to challenges such as broadband availability, financial constraints, and evolving policies. Telehealth must be carefully optimized to address infrastructure gaps and ensure equitable access across rural and urban areas.

The use of dated RUCA codes and ZCTA-level internet speed data introduces limitations in assessing connectivity and geographic classifications, as changes in infrastructure over the last decade may not be adequately captured. These outdated measures could misclassify areas and underestimate connectivity, affecting conclusions about disparities in telehealth access.

Finally, the retrospective design inherently limits causal inferences, as unmeasured variables, such as disease severity, referral patterns, and insurance coverage, may introduce residual confounding. Telehealth encounters might have been selectively offered to patients with less severe conditions or used as a precursor to in-person services, complicating causal interpretation. Prospective studies and randomized trials are needed to better understand the directional relationships between telehealth use and clinical outcomes.

Despite these limitations, our findings provide valuable insights into patterns of telehealth use in cancer care and highlight opportunities for targeted engagement in digital health. Telehealth has emerged as a key tool for improving access to specialized care, increasing efficiency, and enhancing convenience, particularly for patients navigating complex treatments or survivorship. Addressing disparities tied to socioeconomic factors, digital literacy, and geographic location will be crucial to ensuring telehealth delivers equitable care while reducing the burden on patients and caregivers throughout the cancer care continuum [7,16-18,48-50,59].

## Supplementary material

10.2196/79956 Multimedia Appendix 1 List of telehealth-eligible services and associated codes, patient characteristics, patterns of telehealth utilization, association with key healthcare delivery outcomes, model performance, and geographic distribution.

## ABBREVIATIONS

ACG: Adjusted Clinical Groups system
ADI: Area Deprivation Index
CCI: Charlson Comorbidity Index
ED: emergency department
EDC: expanded diagnostic cluster (A diagnosis category based on the ACG system)
EHR: electronic health record
ICD-10-CM: International Classification of Diseases, Tenth Revision, Clinical Modification
ISP: internet service provider
JHHS: Johns Hopkins Health System
PPV: positive predictive value
RUB: Resource Utilization Band (a morbidity stratifier based on the ACG system)
RUCA: Rural-Urban Commuting Area
ZCTA: ZIP Code Tabulation Area
