# Supplementary material for: Patterns of Telehealth Use Across the Cancer Care Continuum and Assessment of Patient and Geographic Factors Associated With Key Healthcare Outcomes: Retrospective Study
Source: JMIR Cancer. 2025 Oct 30;11:e79956. doi: 10.2196/79956 (PMC12574977; doi:10.2196/79956)
Supplement: Multimedia Appendix 1 [file cancer-v11-e79956-s001.docx]

**SUPPLEMENTAL MATERIALS**

| **Supplement Table 1. A List of Telehealth-Eligible Services and Associated Codes.** | | |
| --- | --- | --- |
| **Modality** | **CPT/HCPCS** | **Description** |
| Remote monitoring | 99457 | REMOTE PHYSIOLOGIC MONITORING TREATMENT MANAGEMENT SERVICES, HEALTH CARE PROFESSIONAL TIME IN A CALENDAR MONTH REQUIRING INTERACTIVE COMMUNICATION WITH THE PATIENT/CAREGIVER; FIRST 20 MINUTES |
|  | 99458 | REMOTE PHYSIOLOGIC MONITORING TREATMENT MANAGEMENT SERVICES, HEALTH CARE PROFESSIONAL TIME IN A CALENDAR MONTH REQUIRING INTERACTIVE COMMUNICATION WITH THE PATIENT/CAREGIVER; EACH ADDITIONAL 20-MINUTE |
| Telephone | 98966 | TELEPHONE ASSESSMENT AND MANAGEMENT SERVICE, 5-10 MINUTES OF MEDICAL DISCUSSION |
|  | 98967 | TELEPHONE ASSESSMENT AND MANAGEMENT SERVICE, 11-20 MINUTES OF MEDICAL DISCUSSION |
|  | 98968 | TELEPHONE ASSESSMENT AND MANAGEMENT SERVICE, 21-30 MINUTES OF MEDICAL DISCUSSION |
|  | 99441 | PHYSICIAN TELEPHONE PATIENT SERVICE, 5-10 MINUTES OF MEDICAL DISCUSSION |
|  | 99442 | PHYSICIAN TELEPHONE PATIENT SERVICE, 11-20 MINUTES OF MEDICAL DISCUSSION |
|  | 99443 | PHYSICIAN TELEPHONE PATIENT SERVICE, 21-30 MINUTES OF MEDICAL DISCUSSION |
| Video supported | G0508 | TELEHEALTH CONSULTATION, CRITICAL CARE, INITIAL, PHYSICIANS TYPICALLY SPEND 60 MINUTES COMMUNICATING WITH THE PATIENT AND PROVIDERS VIA TELEHEALTH |
|  | G0509 | TELEHEALTH CONSULTATION, CRITICAL CARE, AND SUBSEQUENT, PHYSICIANS TYPICALLY SPEND 50 MINUTES COMMUNICATING WITH THE PATIENT AND PROVIDERS VIA TELEHEALTH |
|  | G0425 | TELEHEALTH CONSULTATION, EMERGENCY DEPARTMENT, OR INITIAL INPATIENT, TYPICALLY 30 MINUTES COMMUNICATING WITH THE PATIENT VIA TELEHEALTH |
|  | G0426 | TELEHEALTH CONSULTATION, EMERGENCY DEPARTMENT, OR INITIAL INPATIENT, TYPICALLY 50 MINUTES COMMUNICATING WITH THE PATIENT VIA TELEHEALTH |
|  | G0427 | TELEHEALTH CONSULTATION, EMERGENCY DEPARTMENT, OR INITIAL INPATIENT, TYPICALLY 70 MINUTES OR MORE COMMUNICATING WITH THE PATIENT VIA TELEHEALTH |
| **Name** | **Modifiers** | **Description** |
| Video-Supported telehealth | GT | Via interactive audio and video telecommunications systems |
| Video-Supported telehealth | 95 | Synchronous telemedicine service rendered via a real-time interactive audio and video telecommunications system |
| **Name** | **POS code** | **Description** |
| Telehealth-not specified | 02 | The location where health services and health-related services are provided or received, through a telecommunication system |
| CPT: Current Procedural Terminology, HCPCS: Healthcare Common Procedure Coding System | | |

| **Supplement Table 2. Patient Characteristics of the Study Cohort by Quartiles of Telehealth Utilization at the 5-digit Zip Code Tabulation Area Level.**^a^ | | | | | | |
| --- | --- | --- | --- | --- | --- | --- |
| **Patient Characteristics** | **Maryland ZCTA** | | | | **Other ZCTA** | **Total** |
|  | **Q1** | **Q2** | **Q3** | **Q4** |  |  |
| **Total Patients** | 31,295 | 27,580 | 16,711 | 5,741 | 43,647 | 124,974 |
| **Age** | | | | | | |
|  | 62.0 (17.3) | 62.6 (16.7) | 61.4 (16.5) | 61.5 (16.175) | 60.9 (17.51) | 61.7 (17.077) |
| **Sex** | | | | | | |
| Female | 19,862 (63.5%) | 17,187 (62.3%) | 9,923 (59.4%) | 3,104 (54.1%) | 22,614 (51.8%) | 72,690 (58.2%) |
| Male | 11,430 (36.5%) | 10,386 (37.7%) | 6,785 (40.6%) | 2,633 (45.9%) | 20,970 (48%) | 52,204 (41.8%) |
| **Race** | | | | | | |
| African American | 9,355 (29.9%) | 5,206 (18.9%) | 3,824 (22.9%) | 713 (12.4%) | 4,444 (10.2%) | 23,542 (18.8%) |
| Asian | 2,167 (6.9%) | 1,506 (5.5%) | 438 (2.6%) | 107 (1.9%) | 1,691 (3.9%) | 5,909 (4.7%) |
| Caucasian | 16,928 (54.1%) | 18,751 (68%) | 11,528 (69%) | 4,605 (80.2%) | 31,381 (71.9%) | 83,193 (66.6%) |
| Other/Unknown | 2,845 (9.1%) | 2,117 (7.6%) | 921 (5.5%) | 316 (5.5%) | 6,131 (14.0%) | 12,330 (9.8%) |
| **Social Needs** | | | | | | |
| Have at least one social need | 5,601 (18.3%) | 4,698 (17.4%) | 2,276 (14%) | 612 (11%) | 2,606 (6.3%) | 15,793 (13%) |
| **Clinical Characteristics** | | | | | | |
| Average count of chronic conditions | 4.3 (3.9) | 4.2 (4.0) | 3.7 (3.5) | 3.4 (3.3) | 2.2 (2.5) | 3.4 (3.5) |
| Average count of medications | 18.7 (13.9) | 18.4 (13.6) | 17.7 (13.5) | 18.0 (14.1) | 15.1 (13.3) | 17.2 (13.7) |
| Count CCI cancer diagnosis | 20,030 (65.3%) | 17,851 (66%) | 11,150 (68.4%) | 4,105 (73.4%) | 29,666 (71.4%) | 82,802 (68.3%) |
| Count CCI metastatic cancer diagnosis | 9,193 (30%) | 8,005 (29.6%) | 5,201 (31.9%) | 1,914 (34.2%) | 12,012 (28.9%) | 36,325 (30%) |
| Average weighted CCI | 5.4 (4.8) | 5.1 (4.6) | 5.0 (4.5) | 5.1 (4.5) | 4.2 (4.0) | 4.9 (4.5) |
| Average unscaled ACG risk | 4.3 (4.7) | 4.1 (4.6) | 3.6 (4.3) | 3.4 (4.2) | 2.4 (3.4) | 3.5 (4.3) |
| Count with 1+ hospitalization | 12,406 (39.6%) | 9,439 (34.2%) | 5,011 (30%) | 1,754 (30.6%) | 9,320 (21.4%) | 37,930 (30.4%) |
| Count with 1+ emergency visits | 10,491 (33.5%) | 6,419 (23.3%) | 1,972 (11.8%) | 510 (8.9%) | 4,842 (11.1%) | 24,234 (19.4%) |
| Count with RUB4-5 | 16,829 (54.8%) | 14,110 (52.1%) | 7,683 (47.1%) | 2,451 (43.9%) | 12,085 (29.1%) | 53,158 (43.9%) |
| Count any telehealth utilization | 17,676 (57.6%) | 16,238 (60%) | 9,913 (60.8%) | 3,521 (63%) | 17,442 (42%) | 64,790 (53.5%) |
| Count cancer-related telehealth utilization | 8,345 (27.2%) | 8,783 (32.5%) | 6,116 (37.5%) | 2,539 (45.4%) | 12,386 (29.8%) | 38,169 (31.5%) |
| ^a^ Areas where the sample count is < 11 patients are removed from the sample. The quartiles contain an equal number of zip codes and are categorized from the zip codes in Maryland containing the lowest proportion of residents observed to have cancer-related telehealth services at JHHS (Q1) to the highest number (Q4). The out-of-state zip codes (surrounding states or far away) are all put together in a separate group. This category also includes patients missing the zip code information.  ACG: Adjusted Clinical Group, CCI: Charlson Comorbidity Index, JHHS: Johns Hopkins Health System, RUB: Resource Utilization Band, ZCTA: 5-digit Zip Code Tabulation Area. | | | | | | |

| **Supplement Table 3. Assessing the Concurrent Year Cancer-related Telehealth Utilization for Patients Receiving Cancer Care at Johns Hopkins Health System: Adjusted Odds Ratios Associated with Key Patient or Geographic Factors**^a^ | |
| --- | --- |
| **Key Factor** | **Cancer-Related Telehealth Utilization** |
| **Demographic Characteristics** | |
| Age–Years | 0.993 (0.993:0.994) |
| Sex–Male (ref: female) | 1.127 (1.101:1.154) |
| English Proficiency (need for an interpreter) | 0.601 (0.549:0.657) |
| Any Social Needs | 0.851 (0.811:0.894) |
| **Clinical Characteristics**^b^ | |
| Chronic Conditions Count | 1.05 (1.045:1.054) |
| Medication Count | 1.034 (1.033:1.035) |
| Total Medication Gaps | 0.931 (0.921:0.942) |
| Outpatient Visits Count | 1.026 (1.026:1.027) |
| **Charlson Comorbidity Index-defined Cancer Diagnosis** | |
| Cancer Diagnosis | 2.503 (2.415:2.593) |
| Metastatic Cancer Diagnosis | 1.529 (1.491:1.568) |
| **Geospatial Characteristics** | |
| Year | 1.2 (1.194:1.206) |
| National Rank ADI | 0.991 (0.99:0.992) |
| Primary RUCA^c^ | 1.108 (1.092:1.125) |
| COVID-19 Prevalence^d^ | 1.001 (0.998:1.003) |
| Count of Cable ISPs per ZCTA | 0.99 (0.97:1.009) |
| Max Cable Download Speed in Mbps | 1.6 (1.217:2.104) |
| Max Cable Upload Speed in Mbps | 0.688 (0.642:0.738) |
| ^a^ The Odds Ratio (95% Confidence Intervals) is presented for each variable included in the model and for the concurrent year outcome. The model includes a CCI-defined cancer diagnosis to assess how the high-level information regarding the severity of cancer (defined through the CCI variable) would impact the use of cancer-related telehealth services.  ^b^ These clinical measures are derived from the Johns Hopkins ACG System Version 12.0.^22^  ^c^ The RUCA primary categories are ordinal, ranging from 1 to 10 in order of increasing rurality. We treat the primary categories as continuous values for these analyses to simplify the interpretation of linear effects in predictive modeling.  ^d^ Cumulative prevalence per 100K residences through February 2022.  ACG: ACG: Adjusted Clinical Group, ADI: Area Deprivation Index, CCI: Charlson Comorbidity Index, ISP: Internet Service Provider, Mbps: megabits per second, RUCA: Rural-Urban Commuting Area, ZCTA: 5-digit Zip Code Tabulation Area. | |

| **Supplement Table 4. Assessing the Concurrent Year Healthcare Outcomes for Patients Receiving Cancer Care at Johns Hopkins Health System: Adjusted Odds Ratios Associated with Key Patient or Geographic Factors**^a^ | | | |
| --- | --- | --- | --- |
| **Key Factor** | **Emergency Room Visit**^b^ | **Hospitalization**^b^ | **Expected Future Utilization of Higher Cost**^b^ |
| **Telehealth Utilization** | | | |
| Any Cancer-related Telehealth During the Year | 0.916 (0.884:0.948) | 0.83 (0.799:0.863) | 1.146 (1.111:1.181) |
| **Demographic Characteristics** | | | |
| Age–Years | 0.996 (0.995:0.997) | 0.986 (0.985:0.987) | 0.993 (0.992:0.994) |
| Sex–Male (ref: female) | 0.883 (0.855:0.911) | 1.499 (1.448:1.552) | 1.158 (1.125:1.191) |
| English Proficiency (need for an interpreter) | 1.553 (1.42:1.698) | 2.798 (2.558:3.061) | 1.548 (1.425:1.68) |
| Any Social Needs | 1.797 (1.711:1.887) | 1.479 (1.405:1.556) | 1.823 (1.725:1.927) |
| **Clinical Characteristics**^b^ | | | |
| Chronic Conditions Count | 1.06 (1.054:1.066) | 1.259 (1.251:1.266) | 2.244 (2.226:2.263) |
| Medication Count | 1.052 (1.051:1.054) | 1.219 (1.216:1.221) | 1.102 (1.1:1.103) |
| Total Medication Gaps | 1.057 (1.045:1.07) | 0.894 (0.883:0.905) | 0.931 (0.918:0.944) |
| Outpatient Visits Count | 1.008 (1.007:1.008) | 0.99 (0.989:0.991) | 1.021 (1.02:1.022) |
| **Charlson Comorbidity Index-defined Cancer Diagnosis** | | | |
| Cancer Diagnosis | 0.675 (0.651:0.7) | 0.806 (0.77:0.844) | 1.566 (1.506:1.628) |
| Metastatic Cancer Diagnosis | 1.045 (1.008:1.083) | 1.776 (1.71:1.846) | 0.962 (0.932:0.992) |
| **Geospatial Characteristics** | | | |
| Year | 0.969 (0.961:0.977) | 0.906 (0.898:0.913) | 1.034 (1.026:1.042) |
| National Rank ADI | 1.008 (1.001:1.015) | 1.005 (1.004:1.006) | 1.005 (1.004:1.006) |
| Primary RUCA^c^ | 0.409 (0.356:0.47) | 0.906 (0.879:0.935) | 0.926 (0.904:0.947) |
| COVID-19 Prevalence^d^ | 0.722 (0.379:1.376) | 0.99 (0.985:0.994) | 0.999 (0.996:1.002) |
| Count of Cable ISPs per ZCTA | 1.232 (1.202:1.263) | 1.072 (1.042:1.103) | 1.003 (0.979:1.027) |
| Max Cable Download Speed in Mbps | 1.971 (1.041:3.731) | 0.828 (0.526:1.304) | 1.119 (0.772:1.623) |
| Max Cable Upload Speed in Mbps | 1.09 (0.959:1.238) | 0.843 (0.759:0.935) | 1.049 (0.958:1.147) |
| ^a^ The Odds Ratio (95% Confidence Intervals) is presented for each variable included in the model and for each concurrent year outcome. The model includes a CCI-defined cancer diagnosis to assess how the high-level information regarding the severity of cancer (defined through the CCI variable) would impact the use of cancer-related telehealth services.  ^b^ These clinical measures are derived from the Johns Hopkins ACG System Version 12.0. Expected Future Utilization of Higher Cost presents the Resource Utilization Band from the ACG system in the following categories: (1) Healthy users, (2) Low resource utilization, (3) Moderate resource utilization, (4) High resource utilization, (5) Very high resource utilization.^22^  ^c^ The RUCA primary categories are ordinal, ranging from 1 to 10 in order of increasing rurality. We treat the primary categories as continuous values for these analyses to simplify the interpretation of linear effects in predictive modeling.  ^d^ Cumulative prevalence per 100K residences through February 2022.  ACG: Adjusted Clinical Group, ADI: Area Deprivation Index, CCI: Charlson Comorbidity Index, ISP: Internet Service Provider, Mbps: megabits per second, RUCA: Rural-Urban Commuting Area, ZCTA: 5-digit Zip Code Tabulation Area. | | | |

| **Supplement Table 5. Model Performance Metrics for Assessing the Concurrent Year Cancer-related Telehealth Utilization and Healthcare Utilization Outcomes for Patients Receiving Cancer Care Between 2019-2023.** | |
| --- | --- |
| **Area Under the Receiver Operating Characteristic Curve** | |
| Cancer-related Telehealth Utilization ^a^ | 0.797 |
| Emergency room visit^b^ | 0.817 |
| Hospitalization^b^ | 0.958 |
| Elevated (4-5) Resource Utilization Band^b,c^ | 0.968 |
| **Area Under the Precision-Recall Curve** | |
| Cancer-related Telehealth Utilization^a^ | 0.342 |
| Emergency room visit^b^ | 0.216 |
| Hospitalization^b^ | 0.753 |
| Elevated (4-5) Resource Utilization Band^b,c^ | 0.873 |
| **Positive Predictive Value** | |
| Cancer-related Telehealth Utilization^a^ | 0.557 |
| Emergency room visit^b^ | 0.358 |
| Hospitalization^b^ | 0.739 |
| Elevated (4-5) Resource Utilization Band^b,c^ | 0.825 |
| **Sensitivity** | |
| Cancer-related Telehealth Utilization^a^ | 0.132 |
| Emergency room visit^b^ | 0.047 |
| Hospitalization^b^ | 0.599 |
| Elevated (4-5) Resource Utilization Band^b,c^ | 0.721 |
| ^a^ The model consists of patient age, sex, need for interpreter services, presence of any social need, counts of chronic conditions, medications, total medication gaps, outpatient visits count, CCI-defined cancer or metastatic cancer diagnoses, and all ZCTA-level geospatial features.  ^b^ The model consists of patient age, sex, need for interpreter services, presence of any social need, counts of chronic conditions, medications, total medication gaps, outpatient visits count, CCI-defined cancer or metastatic cancer diagnoses, a flag for whether the patient received cancer-related telehealth services, and all ZCTA-level geospatial features.  ^c^ Resource Utilization Band is derived from the Johns Hopkins ACG System Version 12.0. and represents expected future utilization based on current morbidities. It is presented in the following categories: (1) Healthy users, (2) Low resource utilization, (3) Moderate resource utilization, (4) High resource utilization, (5) Very high resource utilization.^22^  ACG: Adjusted Clinical Group, CCi: Charlson Comorbidity Index, ZCTA: 5-digit Zip Code Tabulation Area. | |

**Supplement Figure 1. Mapped Quartile for Cancer-related Telehealth Utilization in the Study Cohort for the Full Observation Period by 5-digit Zip Code Tabulation Area.**^a^


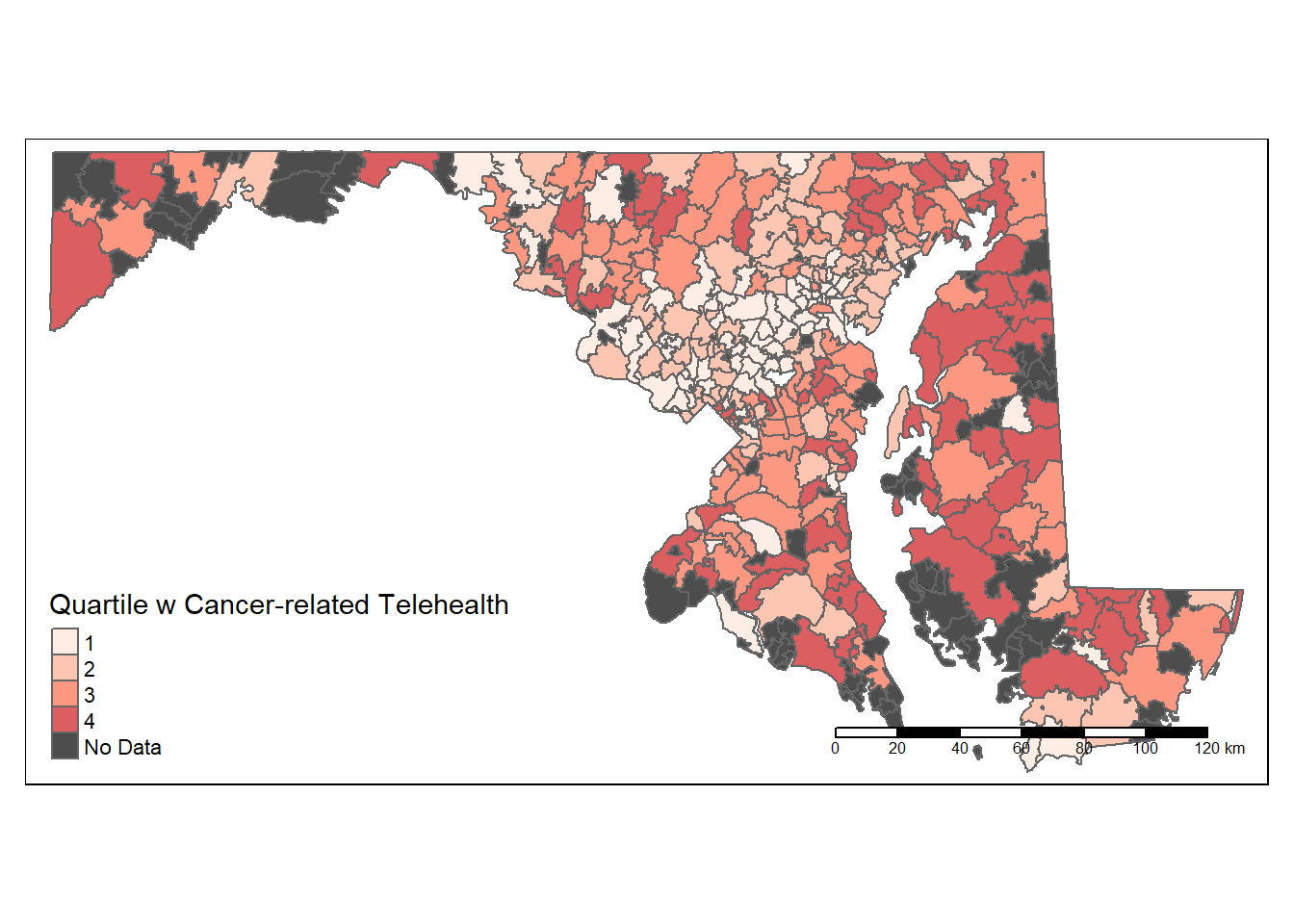


^a^ Traces filled in as black are suppressed due to counts < 11.

**Supplement Figure 2. Correlation Matrix for Geospatial Characteristics using 5-digit Zip Code Tabulation Area.**^a^


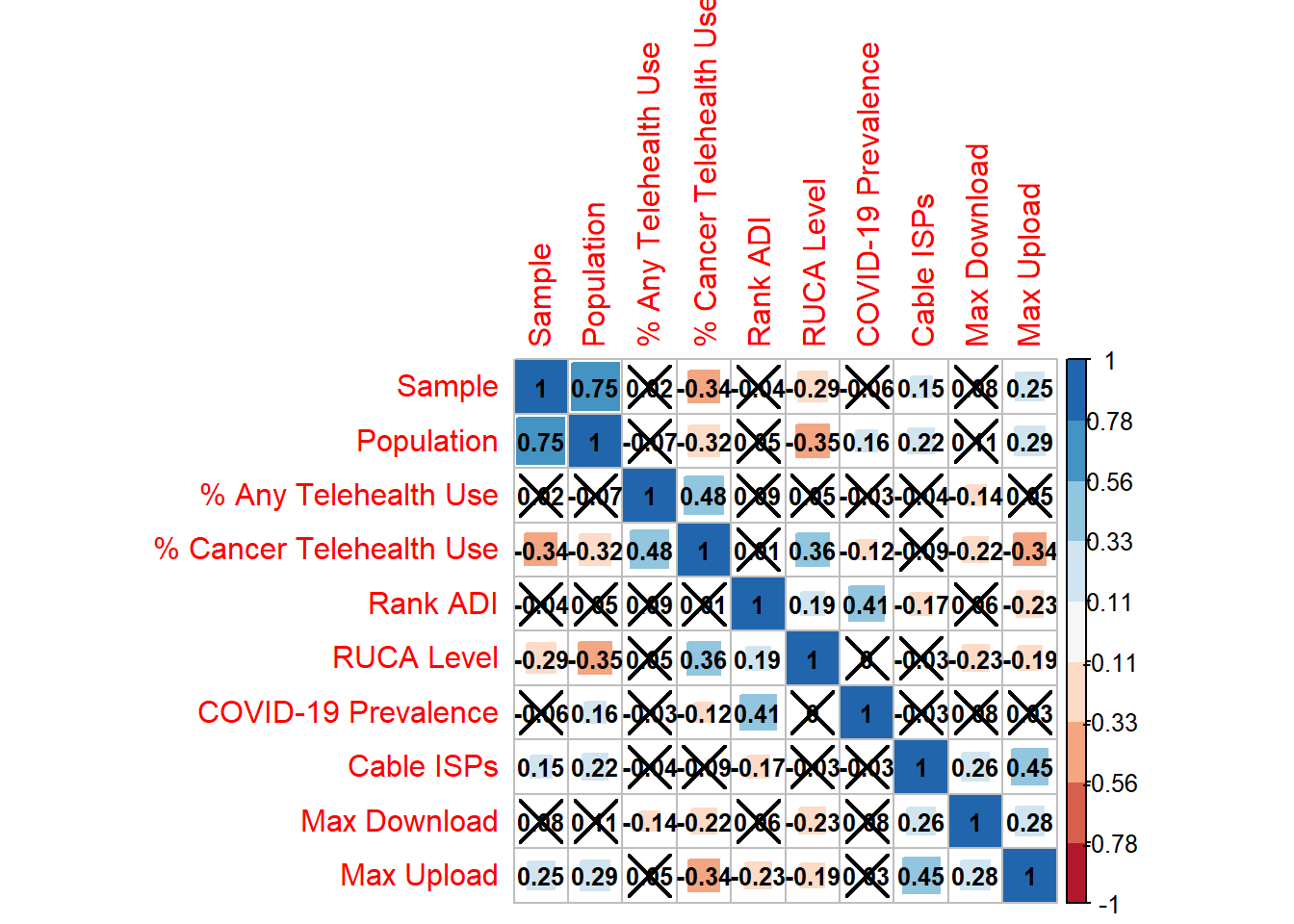


^a^ Areas where the sample count is < 11 patients are removed from the sample.

Max Download presents a maximum cable download speed in Mbps. Max Upload presents a maximum cable upload speed in Mbps.

ADI: Area Deprivation Index, ISP: Internet Service Provider, Mbps: megabits per second, RUCA: Rural-Urban Commuting Area, ZCTA: 5-digit Zip Code Tabulation Area.
